# Association between sleep duration and obesity in patients with type 2 diabetes: A longitudinal study

**DOI:** 10.1111/dme.70051

**Published:** 2025-04-17

**Authors:** Esraa A. Makhdom, Alisha Maher, Ryan Ottridge, Mathew Nicholls, Asad Ali, Brendan G. Cooper, Ramzi A. Ajjan, Srikanth Bellary, Wasim Hanif, Fahmy Hanna, David Hughes, Vijay Jayagopal, Rajni Mahto, Mayank Patel, James Young, Ananth U. Nayak, Mimi Z. Chen, Julie Kyaw‐Tun, Susana Gonzalez, Ravikanth Gouni, Anuradhaa Subramanian, Nicola J. Adderley, Abd A. Tahrani

**Affiliations:** ^1^ Department of Metabolism and Systems Science University of Birmingham Birmingham UK; ^2^ Department of Respiratory Care Imam Abdulrahman Bin Faisal University Dammam Saudi Arabia; ^3^ Centre for Endocrinology, Diabetes and Metabolism, Birmingham Health Partners Birmingham UK; ^4^ Birmingham Clinical Trials Unit University of Birmingham Birmingham UK; ^5^ University Hospitals Coventry and Warwickshire NHS Trust Coventry UK; ^6^ University Hospitals of Birmingham NHS Foundation Trust Birmingham UK; ^7^ Leeds Institute of Cardiovascular and Metabolic Medicine University of Leeds Leeds UK; ^8^ Aston University Birmingham UK; ^9^ University Hospitals of North Midlands NHS Trust Stoke on Trent UK; ^10^ University Hospitals of Derby & Burton NHS Trust Derby UK; ^11^ York Teaching Hospital NHS FT York UK; ^12^ South Warwickshire NHS Foundation Trust South Warwickshire UK; ^13^ University Hospital Southampton NHS FT Southampton UK; ^14^ Royal Wolverhampton Hospitals NHS Trust Wolverhampton UK; ^15^ St George's University Hospitals NHS FT London UK; ^16^ Calderdale and Huddersfield NHS FT Huddersfield UK; ^17^ Bradford Teaching Hospitals NHS FT Bradford UK; ^18^ Nottingham University Hospitals NHS Trust Nottingham UK; ^19^ Department of Applied Health Science University of Birmingham Birmingham UK; ^20^ National Institute for Health and Care Research Birmingham Biomedical Research Centre Birmingham UK

**Keywords:** body mass index, sleep duration, type 2 diabetes, waist circumference

## Abstract

**Background:**

Obesity is prevalent in patients with type 2 diabetes (T2D) and negatively impacts diabetes outcomes. While studies in the general population have established a link between sleep duration and obesity, this relationship in T2D remains unclear.

**Objectives:**

To assess the association between sleep duration and adiposity in patients with T2D.

**Methods:**

This prospective study of adults enrolled in the SLEEP T2D study from 13 UK NHS Trusts. Sleep duration was self‐reported using the Pittsburgh Sleep Quality Index (PSQI) and categorized as short (≤ 6 h/ night), long (> 9 h/night) or (normal > 6‐9 h/night). Adiposity was assessed using body mass index (BMI) and waist circumference.

**Results:**

Among 229 patients (61% male, mean age 61.2 (±11.7) years, 63.7% with BMI ≥ 30 kg/m^2^). At baseline, sleep duration negatively correlated with BMI (*r* = −0.27, *p* < 0.001) and waist circumference (*r* = −0.25, *p* = 0.001). After adjusting for potential confounders in different models, short sleep duration was associated with higher BMI (β = −1.01; *p* = 0.006) and waist circumference (β = −1.91; *p* = 0.01). Following a median follow‐up of 26.5 months, short sleep at baseline was associated with a 5% or more gain in BMI (adjusted OR 10.03; 95% CI 1.55–64.84; *p* = 0.01).

**Conclusion:**

Short sleep duration is associated with higher adiposity measures (BMI and waist circumference) and weight gain in patients with T2D. Addressing sleep duration may reduce the burden of obesity in T2D, and future studies in this area are warranted.

What's new

- The objective of this study is to assess the longitudinal relationship between sleep duration and various adiposity measures in patients with T2D.
- The main aim is to determine whether shorter sleep duration is associated with weight gain in patients with T2D.
- The findings suggest that short sleep duration is significantly associated with increased adiposity measures in patients with T2D and acts as a risk factor for weight gain.
- Clinicians managing patients with T2D and obesity should incorporate sleep duration as a critical factor in treatment and prevention strategies.

## 1 INTRODUCTION

Obesity and type 2 diabetes (T2D) are strongly linked, with over 90% of individuals diagnosed with T2D also exhibiting overweight or obesity.^1^ Obesity is a major risk factor for the onset and progression of T2D.^1^ Consequently, the global prevalence of T2D and obesity has increased simultaneously. In 2022, 16% of the adult global population was living with obesity, and this figure is expected to rise.^2^ According to the International Diabetes Federation, approximately 463 million people had T2D in 2019, which is predicted to reach around 700 million by 2045.^3^ Excess body weight adversely impacts individual survival, cardiometabolic health, and physical and mental well‐being and imposes significant costs on the healthcare system and society at large.^4^ Similarly, T2D is associated with an increased risk of mortality, cardiovascular disease, microvascular complications and elevated healthcare costs.^5^ Hence, there is an urgent need to address obesity in patients with T2D, as this is likely to have a significant positive effect on people's health and healthcare system costs.^6^

To alleviate the burden of obesity in patients with T2D, it is essential to identify modifiable factors that might impact weight. One such factor could be sleeping duration. Sleep disorders are more prevalent in patients with T2D compared to the general population.^7^ Alongside the rise in obesity and T2D prevalence, inadequate sleep has become one of the significant public health risks widespread across all age groups.^8^ Despite the American Academy of Sleep Medicine (AASM) recommendation for sufficient sleep across all ages to promote a healthy life,^9^ recent studies have shown a decline in average sleep duration due to changes in modern lifestyles.^10^ In 2014, short sleep (< 7 h/night) affected more than 35% of United States (U.S.) adults, leading the U.S. Centres for Disease Control and Prevention (CDC) to declare short sleep duration a public epidemic.^11^ Short sleep duration has been linked to various adverse outcomes, including cardiovascular disease and metabolic diseases such as T2D and obesity.^12^ Hence, optimising sleep duration might contribute to reducing adverse health outcomes.

The relationship between sleep duration, obesity and T2D is likely bidirectional.^13^ Several systematic reviews have shown that short sleep duration (and in some long sleep) was associated with an increased risk of obesity and T2D.^14^ Even though the links between sleep duration and obesity are well studied in the general population, there is no data about this relationship in people with T2D, and there is little data about the relationship between sleep duration and body fat distribution. Therefore, in this study, we aimed to assess the relationship between sleep duration and adiposity measures in patients with T2D cross‐sectionally and longitudinally. We hypothesised that short sleep duration is associated with increased adiposity measures in a patient with T2D. We further hypothesised that the presence of short sleep duration contributes to weight gain.

## 2 METHODS

The data for this project are derived from the SLEEP T2D study, an observational cohort study investigating the feasibility of continuous positive airway pressure (CPAP) randomized control trials (RCTs) in a subpopulation with obstructive sleep apnea (OSA). The study protocol and the RCT results have already been published.^15^, ^16^ Here, we report findings from the observational study.

### 2.1 Study design

In this longitudinal analysis, we incorporated baseline data from all study participants, irrespective of their enrollment in the RCT or not, to explore the relationship between sleep duration and adiposity measures over a 2‐year follow‐up period. The project was approved by the National Research Ethics Committee West Midlands—The Black Country, reference 18/WM/0070 and conducted in accordance with the recommendations guiding physicians in biomedical research involving human subjects, as adopted by the 18th World Medical Association (WMA) General Assembly in Helsinki, Finland, 1964, and amended by the 48th WMA General Assembly in Somerset West, Republic of South Africa, 1996.^15^

### 2.2 Setting and Participants

Patients were consecutively recruited from diabetes outpatient departments across 13 UK National Health Services (NHS) Trusts (Appendix S1 in the online supplement file). All participants provided informed consent. Recruitment spanned from July 2018 to February 2020, concluding early due to the onset of the COVID‐19 pandemic. Study data were collected and managed using REDCap (Research Electronic Data Capture), a secure, web‐based platform designed to streamline and support data collection for research purposes.^17^

Study inclusion criteria.

- 18 years old or above;
- Diagnosed with T2D;
- An eGFR ≥15 mL/min/1.73 m^2^ in the last 12 months.

Study exclusion criteria.

- Diagnosed with type 1 diabetes;
- Diagnosed with OSA, active malignancy or chronic kidney disease for reasons other than diabetes;
- On chemotherapy, immunosuppressant drugs or home oxygen treatment;
- A history of frequent hospital admissions due to respiratory infection;
- Received contrast imaging within the last 2 months;
- Pregnant;
- Planning to have bariatric surgery during the study;
- Demonstrated an inability to follow the study protocol;
- Demonstrate inability to provide informed consent;
- A professional driver, operator of heavy machinery or worker at a high altitude;
- A history of falling asleep while driving in the last 2 years.

### 2.3 Data collection

#### 2.3.1 Sleep assessment

Sleep duration was assessed using the validated Pittsburgh Sleep Quality Index (PSQI), a self‐reported questionnaire designed to assess sleep quality over 1 month. The PSQI comprises 19 self‐rated items that generate seven components, culminating in a global sleep quality score.^18^ The score of the 7 components is added to give values between 0 and 21. Poor sleep quality was defined as PSQI >5.^19^

Sleep duration is one of the key components of the PSQI, where participants report their average sleep duration over the past month. This method has been validated and applied across various populations and clinical settings.^18^ For this analysis, short sleep duration was defined as (≤ 6 h/night), long sleep duration (> 9 h/night)^20^ and a normal sleep duration of (> 6‐9) is recommended by the American Academy of Sleep Medicine (AASM).^21^

OSA was assessed through a home‐based, single overnight cardiorespiratory sleep study utilizing a portable multichannel device (ApneaLink Air, ResMed). The results were interpreted according to the guidelines of the American Academy of Sleep Medicine.^16^ Apnoea was defined as a cessation or ≥ a 90% reduction in airflow for at least seconds.^22^ Hypopnea was defined as ≥ 30% reduction in airflow for ≥ 10 seconds, along with ≥ 4% drop in oxygen saturation.^22^ OSA diagnosis was based on the apnoea‐hypopnoea index (AHI) of greater than events/h.^22^

#### 2.3.2 Adiposity measures assessment

Height was measured with a rigid stadiometer to the nearest 0.1 cm and weight in light indoor clothing to the nearest 0.1 kg.^23^ They were measured while patients were standing in a relaxed position. BMI was calculated as weight divided by height squared (kg/m^2^).^23^

Waist, hip and neck circumferences were measured using an inelastic measuring tape. Waist circumference was measured between the inferior ribcage border and the superior aspect of the iliac crest.^24^ Hip circumference was measured at its widest point over the greater trochanters.^25^ Neck circumference was measured midway of the neck, between the mid‐cervical spine and mid‐anterior neck.

High waist circumference was defined as ≥ 102 cm (40 inches) in males and ≥ 88 cm (35 inches) in females.^26^ The BMI was divided into categories, including underweight or normal weight (< 25 kg/m^2^), overweight (25–29.9 kg/m^2^) and obesity (≥ 30 kg/m^2^). Moreover, BMI gain was defined as a 5% or more increase in BMI from baseline.^27^

#### 2.3.3 General/clinical assessment

The following demographic and biological measures were collected:

- Age, gender, ethnicity, diabetes duration and medication;
- Blood pressure;
- Biochemistry assessment, including eGFR, HbA1c, lipids and urine albumin.

### 2.4 The Impact of the COVID‐19 Pandemic

During the COVID‐19 pandemic, the National Health Service (NHS) and research institutions redirected to support frontline efforts. Consequently, this severely impacted non‐COVID‐19 research projects, including ours. In February 2020, we stopped recruitment to ensure patient and staff safety, continuing follow‐ups and data collection remotely, which led to missing data for some variables and outcomes. The pandemic caused variability in the study end‐visit duration and delayed data retrieval, with some exceeding 2 years. Access restrictions at the trial unit site at the University of Birmingham caused a delay in data entry. To address these challenges, the ethics committee approved all necessary protocol amendments.

### 2.5 Statistical analysis

Data analysis was performed using Stata 17. Data are presented as frequency, mean (SD) or median (IQR) depending on the distribution. Histograms and the Shapiro–Wilk test were employed for normality testing. The independent *t*‐test or Mann–Whitney U test was utilized to compare independent continuous variables based on data distribution, whereas the chi‐squared test was applied to compare categorical variables.

The correlation between sleep duration and adiposity measures as continuous variables was assessed using Pearson or Spearman tests.

To assess the relationship between sleep duration and obesity, we used multiple linear regression. To assess if the baseline short sleep duration was associated with future weight gain, we used a logistic regression. Adiposity measures (BMI and waist circumference) were the outcome variables, whereas sleep duration (exposure) and another potential confounder were independent variables. The selection of variables included in the regression models was guided by biological plausibility and established findings in the literature. Statistical significance was defined as *p* < 0.05. All statistical test conditions/assumptions were adhered to throughout the analysis.

## 3 RESULTS

**FIGURE 1:**
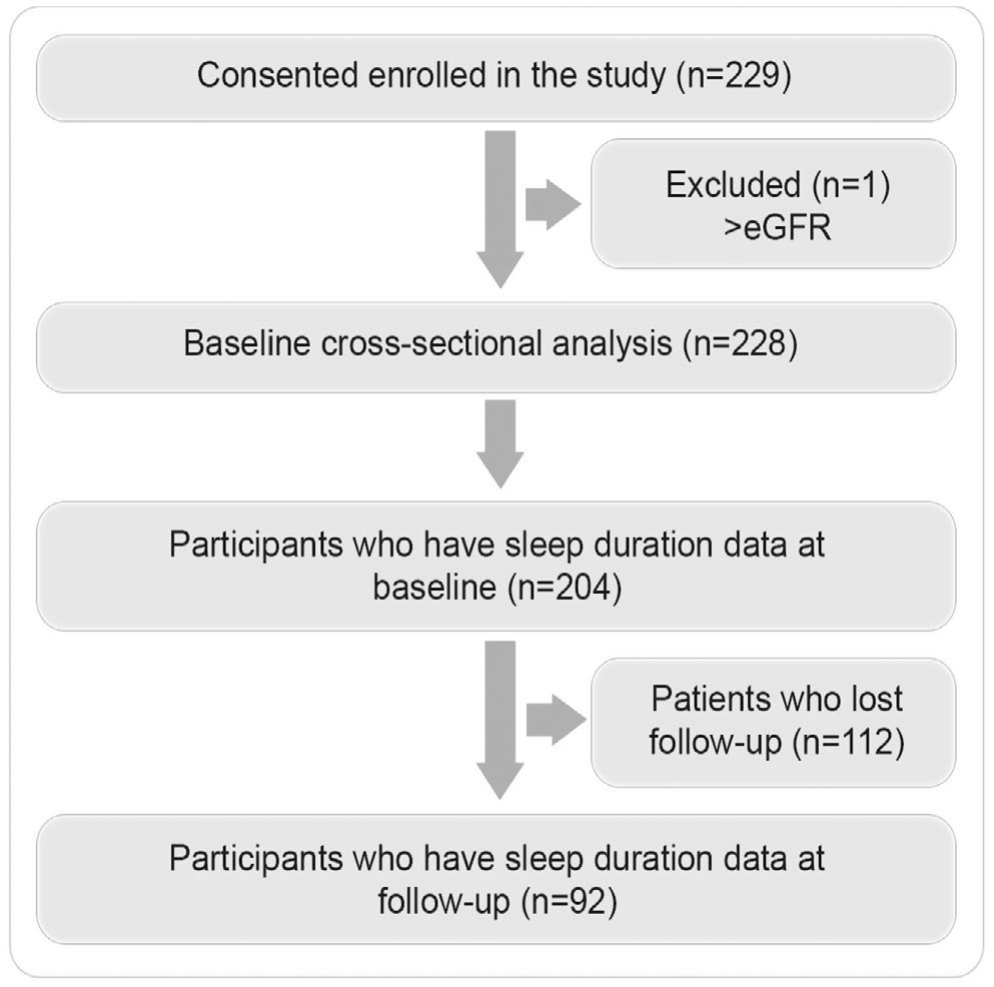
Participants' pathway through the study.

The SLEEP T2D project enrolled a total of 229 patients. The study flow chart is presented in Figure 1. The study population was predominantly male (61.0%, *n* = 122), with a mean age of 61.2 (±11.6) years. The majority of participants were White Europeans, 83.5% (*n* = 168). Most of the population had obesity, 63.7% (*n* = 123), and exhibited reasonable glycaemic control. A summary of the clinical and biochemical baseline characteristics in the total cohort and by sleep duration category is in Table 1.

**TABLE 1:** Patient characteristics at baseline.

	Total (*N* = 204)	Short sleep duration (*N* = 112)	Normal sleep duration (*N* = 88)	*p*‐value (short vs. normal)
Demographics				
Age (years), mean (SD)	61.2 (11.6)	59.8 (10.9)	62.7 (12.5)	**0.03**
Gender: male, *n* (%)	122 (61.0)	59 (53.6)	61 (70.9)	**0.01**
Ethnicity: White, *n* (%)	168 (83.5)	88 (79.3)	77 (88.5)	0.08
Smoking and alcohol status, *n* (%)				
Smoking (ex/current)	117 (57.6)	66 (58.9)	49 (56.3)	0.71
Alcohol (ex/current)	108 (53.5)	49 (43.8)	57 (66.3)	**0.002**
Diabetes duration and medication used				
Diabetes duration (years), Median [IQR]	13 (6–19)	13 (6–19.5)	12 (6–19)	0.96
Insulin: Yes, *n* (%)	105 (51.9)	63 (56.3)	40 (46.5)	0.17
GLP‐1 agonist): Yes, *n* (%)	33 (17.7)	21 (20.0)	11 (13.9)	0.28
Lipid lowering agents (statin): Yes, *n* (%)	141 (71.9)	79 (73.2)	58 (69.1)	0.53
Anti‐hypertensive (ACE inhibitor): Yes, *n* (%)	87 (45.3)	49 (45.4)	37 (45.6)	0.96
Blood pressure (mmHg)				
Blood pressure (Systolic mmHg), mean (SD)	132.9 (15.9)	133.2 (15.9)	133.4 (15.9)	0.86
Blood pressure (diastolic mmHg), Mean (SD)	75.7 (10.6)	74.2 (10. 5)	77.8 (10.5)	**0.03**
Total cholesterol (mmol/L), mean (SD)	4.1 (1.1)	4.2 (1.0)	4.1 (1.1)	0.84
HbA1c (mmol/mol), mean (SD)	65.7 (21.4)	67.1 (20.7)	64.8 (21.5)	0.38
eGFR (mL/min/1.73 m^2^), mean (SD)	82.6 (26.6)	81.9 (29.1)	83.0 (23.1)	0.79
Adiposity measures				
BMI (kg/m^2^), mean (SD)	33.8 (7.9)	35.7 (8.8)	31.7 (5.9)	**0.002**
Obesity (BMI ≥ 30 kg/m^2^), *n* (%)	123 (63.7)	74 (70.5)	49 (57.7)	**0.06**
Waist circumference (cm), mean (SD)	113.9 (15.2)	116.9 (16.2)	109.4 (13.0)	**0.001**
High waist, *n* (%)	135 (66.2)	73 (65.2)	58 (65.9)	0.91
Hip circumference (cm), mean (SD)	114.5 (13.6)	117.6 (15.3)	111.1 (10. 5)	**0.001**
Neck circumference (cm), mean (SD)	41.8 (4.5)	42.3 (4.8)	41.5 (4.2)	0.24

*Note*: Data are presented as median (IQR) or mean (SD). Categorical variables are presented as *n* (%). Analysis was performed using the chi‐squared test for categorical variables, the independent *t*‐test for normally distributed variables, and the Mann–Whitney U test for non‐normally distributed variables.

Abbreviations: eGFR, glomerular filtrate rate; GLP‐1, glucagon‐like peptide 1; HbA1c, glycated haemoglobin.

Compared to patients in the normal sleep group, patients in the short sleep group were younger, heavier and had lower diastolic blood pressure. Although they were prescribed more insulin (56.3% vs. 46.5%) and GLP‐1 receptor agonists (20% vs. 13.9%) than those in the normal sleep group, this difference was not statistically significant. Patients in both groups (short and normal) had similar diabetes durations and total cholesterol levels.

### 3.1 Sleep Duration

A total of 204 patients provided self‐reported sleep duration as a part of the PSQI questionnaire. The mean (SD) sleep duration was 6.30 (1.7) h. Short sleep duration was self‐reported in 54.9% (*n* = 112). Due to the limited number of patients with long sleep duration (*n* = 4), this group was excluded from the analysis based on sleep duration categories but was included when sleep duration was examined as a continuous variable.

### 3.2 Sleep quality

Out of 229 participants enrolled in the SLEEP T2D, 194 reported their baseline data on sleep quality and the total PSQI score. The prevalence of poor sleep was 75.8% (*n* = 147). The clinical and biochemical baseline characteristics of the study population according to sleep quality category are shown in Table S1 in the online supplement file.

In linear regression, sleep quality was significantly associated with BMI (β = 0.57, *p* < 0.0001) and waist circumference (β = 1.20, *p* < 0.0001). In the adjusted analysis, both BMI and waist circumference remained independently associated with sleep quality (β = 0.42, *p* = 0.008 and β = 1.37, *p* < 0.0001, respectively) (Table S2 in online supplement file). The association remained significant after adjusting for OSA, which suggests that higher BMI and waist circumference were associated with a higher PQSI score, indicating poorer sleep quality.

### 3.3 Baseline cross‐sectional analysis of the relationship between sleep duration and adiposity measures

There were significant differences in adiposity measures between patients with short sleep duration and those with normal sleep duration (Table 1). Patients with short sleep duration had higher BMI, waist and hip circumferences than patients with normal sleep duration.

Sleep duration correlated negatively with BMI and waist circumference (r = −0.27, *p* < 0.001 and r = −0.25, *p* = 0.001, respectively) (Figure S1 in online supplement file). Hence, a shorter sleep duration is associated with higher BMI and waist circumference.

### 3.4 Sleep Duration and Adiposity Measure at Baseline: Multivariable Analysis

After adjustment for potential confounders (as detailed in Table 2), shorter sleep duration was associated with higher BMI and waist circumference (Table 2). This association remained significant even after adding the AHI to the model in the case of the BMI. Additionally, using GLP‐1 receptor agonists was associated with a higher waist circumference (β = 7.06: *p* = 0.05) in model 4, which could be attributed to prescribing GLP‐1 receptor analogues in patients with a higher adiposity. Another significant association with adiposity measures was that alcohol was associated with higher waist circumference (*p* < 0.001).

**TABLE 2:** Assessing the association between sleep duration and adiposity measures, including BMI and waist circumference, based on baseline cross‐sectional analysis using multiple linear regression.

Model	*R* ^2^	Coefficient	95% CI	*p*‐value
BMI				
Unadjusted	0.09	−1.47	−211 to −0.83	<0.001
Model^1^	0.18	−1.30	−1.99 to −0.60	<0.001
Model^2^	0.23	−1.17	−1.90 to −0.44	0.002
Model^3^	0.27	−1.01	−1.74 to −0.28	0.006
Model^4^	0.41	−0.74	−1.54 to 0.06	0.07
Waist circumference				
Unadjusted	0.06	−2.36	−3.74 to −0.99	0.001
Model^1^	0.10	−2.37	−3.92 to −0.83	0.003
Model^2^	0.15	−2.35	−3.95 to −0.75	0.004
Model^3^	0.24	−1.91	−3.47 to −0.35	0.01
Model^4^	0.35	−0.92	−2.73 to 0.89	0.31

Abbreviations: BMI, body mass index; CI, confidence interval.

Model^1^ is adjusted for age, ethnicity, gender, and diabetes duration.

Model^2^ is adjusted for age, ethnicity, gender, diabetes duration, insulin use and GLP‐1 receptor use.

Model^3^ is adjusted for age, ethnicity, gender, duration of diabetes, insulin use, GLP‐1 receptor use, smoking status and alcohol use.

Model^4^ is adjusted for age, ethnicity, gender, diabetes duration, insulin use, GLP‐1 receptor use, smoking status, alcohol use and apnoea hypopnea index (AHI).

### 3.5 Sleep duration and BMI: longitudinal analysis

A longitudinal analysis was conducted on a subset of study participants with available baseline and follow‐up data (*n* = 92). Among the 92 patients with follow‐up sleep data, 9 had missing baseline sleep duration information. Of the 37 identified as short sleepers at baseline, 24 remained short sleepers, while 13 transitioned to normal sleep duration. Conversely, of the 44 patients with normal sleep duration at baseline, 11 shifted to short sleep duration, and 33 maintained their normal sleep patterns. Of the 2 patients with long sleep duration, 1 transitioned to normal sleep, while the other remained a long sleeper. The median follow‐up period was 26.5 months (IQR 22.9–31.5). No significant changes in adiposity measures were observed in patients between baseline and study end, regardless of whether patients initially categorized as short or normal sleepers experienced changes in their sleep patterns during the follow‐up period (Table S3 in online supplement file).

Using linear regression, shorter sleep duration at baseline was associated with higher study end BMI and waist circumference despite adjusting for multiple variables, except when the baseline value of the adiposity measure was inserted into the regression model (Table 3).

**TABLE 3:** Assessing the association between baseline sleep duration and the follow‐up adiposity measures, including BMI and waist circumference, using multiple linear regression.

Model	*R* ^2^	Coefficient	95% CI	*p* value
BMI				
Unadjusted	0.13	−1.43	−2.27 to −0.59	0.001
Model^1^	0.24	−1.52	−2.48 to −0.56	0.002
Model^2^	0.29	−1.64	−2.68 to −0.60	0.002
Model^3^	0.36	−1.41	−2.47 to −0.34	0.01
Model^4^	0.85	0.36	−1.54 to 2.27	0.70
Waist circumference				
Unadjusted	0.13	−3.35	−5.34 to −1.36	0.001
Model^1^	0.17	−3.46	−5.79 to −1.13	0.004
Model^2^	0.21	−3.95	−6.48 to −1.43	0.003
Model^3^	0.29	−3.28	−5.87 to −0.70	0.01
Model^5^	0.83	−0.54	−2.20 to 1.10	0.50

Abbreviations: BMI, body mass index; CI, confidence interval.

Model^1^ is adjusted for age, ethnicity, gender and diabetes duration.

Model^2^ is adjusted for age, ethnicity, gender, diabetes duration, insulin use and GLP‐1 receptor use.

Model^3^ is adjusted for age, ethnicity, gender, duration of diabetes, insulin use, GLP‐1 receptor use, smoking status and alcohol use.

Model^4^ is adjusted for age, ethnicity, gender, diabetes duration, insulin use, GLP‐1 receptor use, smoking status and alcohol use, apnoea hypopnea index (AHI) and baseline BMI.

Model^5^ is adjusted for age, ethnicity, gender, diabetes duration, insulin use, GLP‐1 receptor use, smoking status and alcohol use, AHI and baseline WC.

To assess whether short duration at baseline was associated with clinically meaningful weight gain (defined as a BMI increase of 5% or more from baseline), we conducted a logistic regression analysis. After adjusting for relevant confounders, short sleep duration at baseline remained significantly associated with a 5% or more gain in BMI with OR 10.03 (95% CI 1.55–64.84; *p* = 0.01) (Table 4). However, caution is advised when interpreting the OR due to the imbalance in sex distribution.

**TABLE 4:** Association between baseline short sleep duration and weight gain (defined as a 5% or more increase in BMI from baseline).

	Pseudo‐*R* ^2^	OR	95% CI	*p* value
5% gained in BMI				
Unadjusted	0.07	4.30	1.06–17.38	0.04
Model^a^	0.15	8.53	1.42–51.08	0.01
Model^b^	0.16	10.03	1.55–64.84	0.01

^a^ Adjusted for age, diabetes duration, sex, smoking status and alcohol consumption.

^b^ Adjusted for age, diabetes duration, sex, smoking status, alcohol consumption, insulin use and GLP‐1 receptor use.

## 4 DISCUSSION

This is the first report investigating the relationship between sleep duration and adiposity measures in patients with T2D with several novel findings. Short sleep duration is very common among patients with T2D and is associated with greater adiposity at baseline. In addition, shorter sleep was associated with higher adiposity measures at the end of the follow‐up. Short sleep duration was associated with higher chances of weight gain, at least 5%, during the follow‐up. Furthermore, poor sleep quality was associated with higher BMI and waist circumference at baseline.

The findings of this study are consistent with studies in the general population, which also demonstrate an association between short sleep and obesity.^11^, ^20^ Results from a meta‐analysis involving 45 cross‐sectional studies in population‐based studies of adults and children from around the world (604,509 adults and 30,002 children) suggested a strong relationship between short sleep duration and obesity, with an OR 1.55 (1.43–1.68; *p* < 0.0001) in the adult population.^28^ Our findings add to the strength of the evidence linking short sleep duration to obesity by extending the findings to people living with T2D.

The relationship between sleep duration, adiposity, and T2D is complex and multifactorial. Short or inefficient sleep duration can impact energy homeostasis, beta cell function and insulin sensitivity through several pathways. Key mechanisms include hormonal imbalances, such as alterations in the ghrelin‐to‐leptin ratio, which regulates hunger and satiety, as well as changes in brain activity in response to food stimuli and a reduction in glucagon‐like peptide‐1 (GLP‐1) levels. Collectively, these physiological changes might lead to increased appetite and promote weight gain. Additionally, short sleep duration impacts cortisol secretion, resulting in disturbance of the glucose‐insulin metabolism and substrate oxidation and, ultimately, increased risk of obesity.^11^, ^29^ In addition to these metabolic effects, more awake time may allow more eating and fatigue, contributing to lower physical activity and reduced energy expenditure.^30^ The health consequences of chronic sleep deprivation are broad and significant. Short sleep duration has been associated with various significant health outcomes, including mortality,^31^ hypertension, cardiovascular disease (CVD) and stroke,^32^ conditions commonly seen in patients with T2D. This adds further importance to addressing short sleep duration as part of the care delivered to people with T2D.

Despite the fact that short sleep duration is very common and has significant health implications, there are limited resources dedicated to promoting longer sleep duration. Several studies have highlighted the health benefits of increased sleep duration, including improved blood pressure, better glucose levels and reduced craving for high‐calorie foods.^33^ One interventional study on healthy adults indicated that extending sleep improves insulin sensitivity.^34^ Similarly, recent randomised control trials (RCTs) have shown that sleep extension positively influences both blood pressure and insulin regulation.^35^ Additional research has linked increased sleep duration with decreased insulin resistance and reduced appetite.^36^, ^37^ Experimental studies also suggested that short sleep duration enhances the risk of obesity in adults.^38^ In the LIFE study, a two‐phase RCT, participants in the non‐randomized phase experienced an average weight loss of 6.3 kg, with 60% losing at least 4.5 kg. This successful weight loss was associated with increased sleep duration.^39^ Furthermore, bariatric surgery has been shown to improve sleep quality and prolong sleep duration.^40^ These findings collectively highlight the potential health benefits of sleep extension and emphasise the need for additional resources and interventions to increase sleep duration to improve overall health outcomes. However, the potential benefits of sleep extension in patients with T2D need to be examined in appropriately designed studies.

Our study has several strengths and limitations. This is the first study to investigate the longitudinal relationship between sleep duration and adiposity measures, specifically BMI and waist circumference, in patients with T2D. A key strength lies in including a well‐characterized cohort with a comprehensive range of demographics and clinical variables measured, allowing adjustment for a wide range of potential confounders. However, a significant limitation is the smaller sample size available for the longitudinal analysis, largely due to the disruption caused by the COVID‐19 pandemic, limiting the generalizability of our findings. Recruitment occurred from July 2018 until it was halted in February 2020 due to the COVID‐19 pandemic, which might affect the outcome, as individuals underwent lifestyle changes during and after the pandemic. However, the effect of the COVID‐19 pandemic fell outside this analysis's scope.

The data used in this analysis are from the SLEEP T2D study, an observational study with an embedded randomised control trial for a subgroup of participants randomly assigned to CPAP or No CPAP. The study was powered for its primary feasibility outcomes but not for the secondary outcomes, including the association between sleep duration and obesity in patients with T2D.

The association between short sleep duration and adiposity measures remained significant even after adjusting for AHI, a key diagnostic measure for OSA. However, due to the poor CPAP compliance in our study, it was difficult to assess the impact of CPAP on the relationship between sleep duration and adiposity measures in this study. Despite the influence of key confounders such as socio‐economic status and shift work, those factors were not included in the data set due to the descriptive nature of the analysis.

Additionally, there is a reliance on self‐reported sleep duration. Despite that self‐reported sleep duration can introduce bias, self‐reported sleep duration has been widely used.^41^ Several studies have compared self‐reported with measured sleep duration. These studies showed a moderate correlation between self‐reported and measured sleep duration. More importantly, people over‐reported their habitual sleep duration, with the overestimation being more significant among short sleepers than those with a normal sleep length. For example, persons sleeping 5 h over‐reported their sleep duration by 1.2 h and those sleeping 7 h over‐reported by 0.4 h.^42^, ^43^ This suggests that the prevalence of short sleep duration in our sample could be underreported. However, those with short sleep duration in our study population are very likely to have short sleep duration using measured sleep duration. While some of the patients with normal sleep duration in our study (especially those with 6 h of sleep) might be misclassified it is likely that our results remain true.

## 5 CONCLUSION

In conclusion, short sleep duration is associated with increased adiposity measures in patients with T2D. We also found that short sleep duration is very common in patients with T2D; hence, interventional studies are needed to evaluate whether increasing the average amount of sleep might be beneficial and potentially be used as a treatment strategy in patients with T2D to lose weight.

## AUTHOR CONTRIBUTIONS

AAT conceived the study's idea and designed the protocol. EAM carried out the statistical analysis and wrote the first draft of the manuscript. AAT, NJA and AS supervised the study and analysis. All authors reviewed and revised the manuscript.

## FUNDING INFORMATION

This project was funded as part of National Institue for Health Research (NIHR), Clinician Scientist Award (CS‐2013‐13‐029). The views presented in this manuscript are those of the authors and not those of NIHR or the NHS.

## CONFLICT OF INTEREST STATEMENT

All authors declared no conflict of interest except:

- AAT is currently an employee of Novo Nordisk and has shares. The views expressed in this manuscript are those of the author and not Novo Nordisk. Novo Nordisk had no role in this manuscript.
- Srikanth Bellary reports: I have received speaker fees and honoraria from Novo Nordisk, Eli Lilly, Boehringer Ingelheim, and Astra Zeneca.
- Mayank Patel reports: I have received speaker fees from Eli Lilly and Company, Insulate and Astra Zeneca.

## Supporting information


Data S1.

## References

[1] Grant B , Sandelson M , Agyemang‐Prempeh B , Zalin A . Managing obesity in people with type 2 diabetes. Clin Med (Lond). 2021;21(4):e327‐e231. doi:10.7861/clinmed.2021-0370 35192472 PMC8313195

[2] Organization WH . Obesity and Overweight. Accessed June 9, 2021, https://www.who.int/news‐room/fact‐sheets/detail/obesity‐and‐overweight.

[3] Sun H , Saeedi P , Karuranga S , et al. IDF diabetes atlas: global, regional and country‐level diabetes prevalence estimates for 2021 and projections for 2045. Diabetes Res Clin Pract. 2022;183:109119. doi:10.1016/j.diabres.2021.109119 34879977 PMC11057359

[4] Djalalinia S , Qorbani M , Peykari N , Kelishadi R . Health impacts of obesity. Pak J Med Sci. 2015;31(1):239‐242. doi:10.12669/pjms.311.7033 25878654 PMC4386197

[5] Whicher CA , O'Neill S , Holt RIG . Diabetes in the UK: 2019. Diabet Med. 2020;37(2):242‐247. doi:10.1111/dme.14225 31901175

[6] Chan RS , Woo J . Prevention of overweight and obesity: how effective is the current public health approach. Int J Environ Res Public Health. 2010;7(3):765‐783. doi:10.3390/ijerph7030765 20617002 PMC2872299

[7] Schipper SBJ , Van Veen MM , Elders PJM , et al. Sleep disorders in people with type 2 diabetes and associated health outcomes: a review of the literature. Diabetologia. 2021;64(11):2367‐2377. doi:10.1007/s00125-021-05541-0 34401953 PMC8494668

[8] Arora T , Hussain S , Hubert Lam KB , Lily Yao G , Neil Thomas G , Taheri S . Exploring the complex pathways among specific types of technology, self‐reported sleep duration and body mass index in UK adolescents. Int J Obes (Lond). 2013;37(9):1254‐1260. doi:10.1038/ijo.2012.209 23295500

[9] Paruthi S , Brooks LJ , D'Ambrosio C , et al. Consensus statement of the American Academy of sleep medicine on the recommended amount of sleep for healthy children: methodology and discussion. J Clin Sleep Med. 2016;12(11):1549‐1561. doi:10.5664/jcsm.6288 27707447 PMC5078711

[10] Jackson CL , Redline S , Kawachi I , Hu FB . Association between sleep duration and diabetes in black and white adults. Diabetes Care. 2013;36(11):3557‐3565. doi:10.2337/dc13-0777 24026552 PMC3816913

[11] Antza C , Kostopoulos G , Mostafa S , Nirantharakumar K , Tahrani A . The links between sleep duration, obesity and type 2 diabetes mellitus. J Endocrinol. 2021;252(2):125‐141. doi:10.1530/JOE-21-0155 34779405 PMC8679843

[12] Arora A , Pell D , van Sluijs EMF , Winpenny EM . How do associations between sleep duration and metabolic health differ with age in the UK general population? PLoS One. 2020;15(11):e0242852. doi:10.1371/journal.pone.0242852 33227026 PMC7682906

[13] Lucassen EA , Rother KI , Cizza G . Interacting epidemics? Sleep curtailment, insulin resistance, and obesity. Ann N Y Acad Sci. 2012;1264:110‐134. doi:10.1111/j.1749-6632.2012.06655.x 22827862 PMC3418485

[14] Guimaraes KC , Silva CM , Latorraca COC , Oliveira RA , Crispim CA . Is self‐reported short sleep duration associated with obesity? A systematic review and meta‐analysis of cohort studies. Nutr Rev. 2022;80(5):983‐1000. doi:10.1093/nutrit/nuab064 34508648

[15] Antza C , Ottridge R , Patel S , et al. The impact of sleep disorders on microvascular complications in patients with type 2 diabetes (SLEEP T2D): the protocol of a cohort study and feasibility randomised control trial. Pilot Feasibility Stud. 2021;7(1):80. doi:10.1186/s40814-021-00817-z 33752759 PMC7982768

[16] Makhdom EA , Maher A , Ottridge R , et al. The impact of obstructive sleep apnea treatment on microvascular complications in patients with type 2 diabetes: a feasibility randomized controlled trial. J Clin Sleep Med. 2024;20(6):947‐957. doi:10.5664/jcsm.11020 38318821 PMC11145053

[17] Harris RT , Minor BL , Elliott V , et al. The REDCap consortium: building an international community of software platform partners. J Biomed Inform. 2019;95:103208. 31078660 10.1016/j.jbi.2019.103208 PMC7254481

[18] Buysse DJ , Reynolds CF 3rd , Monk TH , Berman SR , Kupfer DJ . The Pittsburgh sleep quality index: a new instrument for psychiatric practice and research. Psychiatry Res. 1989;28(2):193‐213. doi:10.1016/0165-1781(89)90047-4 2748771

[19] Park BK . The Pittsburg sleep quality index (PSQI) and associated factors in middle‐school students: a cross‐sectional study. Child Health Nurs Res. 2020;26(1):55‐63. doi:10.4094/chnr.2020.26.1.55 35004450 PMC8650888

[20] Anic GM , Titus‐Ernstoff L , Newcomb PA , Trentham‐Dietz A , Egan KM . Sleep duration and obesity in a population‐based study. Sleep Med. 2010;11(5):447‐451. doi:10.1016/j.sleep.2009.11.013 20363668 PMC2854876

[21] Watson NF , Badr MS , Belenky G , et al. Recommended amount of sleep for a healthy adult: a joint consensus statement of the American Academy of sleep medicine and Sleep Research Society. Sleep. 2015;38(6):843‐844. doi:10.5665/sleep.4716 26039963 PMC4434546

[22] Grigg‐Damberger MM . The AASM Scoring Manual four years later. J Clin Sleep Med. 2012;8(3):323–332. 22701392 10.5664/jcsm.1928 PMC3365093

[23] Liu J , Tse LA , Liu Z , et al. Predictive values of anthropometric measurements for Cardiometabolic risk factors and cardiovascular diseases among 44 048 Chinese. J Am Heart Assoc. 2019;8(16):e010870. doi:10.1161/JAHA.118.010870 31394972 PMC6759887

[24] Klein S , Allison DB , Heymsfield SB , et al. Waist circumference and cardiometabolic risk: a consensus statement from shaping America's health: Association for Weight Management and Obesity Prevention; NAASO, the Obesity Society; the American Society for Nutrition; and the American Diabetes Association. Am J Clin Nutr. 2007;85(5):1197‐1202. doi:10.1093/ajcn/85.5.1197 17490953

[25] Snijder MB , Dekker JM , Visser M , et al. Associations of hip and thigh circumferences independent of waist circumference with the incidence of type 2 diabetes: the Hoorn study. Am J Clin Nutr. 2003;77(5):1192‐1197. doi:10.1093/ajcn/77.5.1192 12716671

[26] Purnell JQ . In: AB FKR , Blackman MR , eds. Definitions, Classification, and Epidemiology of Obesity. MDText.com, Inc; 2023.

[27] Wing RR , Lang W , Wadden TA , et al. Benefits of modest weight loss in improving cardiovascular risk factors in overweight and obese individuals with type 2 diabetes. Diabetes Care. 2011;34(7):1481‐1486. doi:10.2337/dc10-2415 21593294 PMC3120182

[28] Cappuccio FP , Taggart FM , Kandala NB , et al. Meta‐analysis of short sleep duration and obesity in children and adults. Sleep. 2008;31(5):619‐626. doi:10.1093/sleep/31.5.619 18517032 PMC2398753

[29] Shan Z , Ma H , Xie M , et al. Sleep duration and risk of type 2 diabetes: a meta‐analysis of prospective studies. Diabetes Care. 2015;38(3):529‐537. doi:10.2337/dc14-2073 25715415

[30] Chaput JP , Bouchard C , Tremblay A . Change in sleep duration and visceral fat accumulation over 6 years in adults. Obesity (Silver Spring). 2014;22(5):E9‐E12. doi:10.1002/oby.20701 24420871

[31] Shen X , Wu Y , Zhang D . Nighttime sleep duration, 24‐hour sleep duration and risk of all‐cause mortality among adults: a meta‐analysis of prospective cohort studies. Sci Rep. 2016;6:21480. doi:10.1038/srep21480 26900147 PMC4761879

[32] Itani O , Jike M , Watanabe N , Kaneita Y . Short sleep duration and health outcomes: a systematic review, meta‐analysis, and meta‐regression. Sleep Med. 2017;32:246‐256. doi:10.1016/j.sleep.2016.08.006 27743803

[33] Adkins EC , DeYonker O , Duffecy J , Hooker SA , Baron KG . Predictors of intervention interest among individuals with short sleep duration. J Clin Sleep Med. 2019;15(8):1143‐1148. doi:10.5664/jcsm.7808 31482836 PMC6707060

[34] Leproult R , Deliens G , Gilson M , Peigneux P . Beneficial impact of sleep extension on fasting insulin sensitivity in adults with habitual sleep restriction. Sleep. 2015;38(5):707‐715. doi:10.5665/sleep.4660 25348128 PMC4402666

[35] Hartescu I , Stensel DJ , Thackray AE , et al. Sleep extension and metabolic health in male overweight/obese short sleepers: a randomised controlled trial. J Sleep Res. 2022;31(2):e13469. doi:10.1111/jsr.13469 34459060

[36] So‐Ngern A , Chirakalwasan N , Saetung S , Chanprasertyothin S , Thakkinstian A , Reutrakul S . Effects of two‐week sleep extension on glucose metabolism in chronically sleep‐deprived individuals. J Clin Sleep Med. 2019;1(5):711‐718. doi:10.5664/jcsm.7758 PMC6510689 31053213

[37] Tasali E , Chapotot F , Wroblewski K , Schoeller D . The effects of extended bedtimes on sleep duration and food desire in overweight young adults: a home‐based intervention. Appetite. 2014;80:220‐224. doi:10.1016/j.appet.2014.05.021 24858836 PMC4112413

[38] Hoddy KK , Potts KS , Bazzano LA , Kirwan JP . Sleep extension: a potential target for obesity treatment. Curr Diab Rep. 2020;20(12):81. doi:10.1007/s11892-020-01360-6 33275183 PMC8140617

[39] Elder CR , Gullion CM , Funk KL , DeBar LL , Lindberg NM , Stevens VJ . Impact of sleep, screen time, depression and stress on weight change in the intensive weight loss phase of the LIFE study. Int J Obes (Lond). 2012;36(1):86‐92. doi:10.1038/ijo.2011.60 21448129 PMC3136584

[40] Toor P , Kim K , Buffington CK . Sleep quality and duration before and after bariatric surgery. Obes Surg. 2012;22(6):890‐895. doi:10.1007/s11695-011-0541-8 22101850

[41] Miner B , Stone KL , Zeitzer JM , et al. Self‐reported and actigraphic short sleep duration in older adults. J Clin Sleep Med. 2022;18(2):403‐413. doi:10.5664/jcsm.9584 34338629 PMC8804982

[42] Lauderdale DS , Knutson KL , Yan LL , Liu K , Rathouz PJ . Self‐reported and measured sleep duration: how similar are they? Epidemiology. 2008;19(6):838‐845. doi:10.1097/EDE.0b013e318187a7b0 18854708 PMC2785092

[43] Matthews KA , Patel SR , Pantesco EJ , et al. Similarities and differences in estimates of sleep duration by polysomnography, actigraphy, diary, and self‐reported habitual sleep in a community sample. Sleep Health. 2018;4(1):96‐103. doi:10.1016/j.sleh.2017.10.011 29332687 PMC5771411

