# Supplementary material for: Association between sleep duration and obesity in patients with type 2 diabetes: A longitudinal study
Source: Diabet Med. 2025 Apr 17;42(6):e70051. doi: 10.1111/dme.70051 (PMC12080980; doi:10.1111/dme.70051)
Supplement: Supplementary file 1 — Data S1. [file DME-42-e70051-s001.docx]

# Appendix S1:

**SLEEP T2D Sites:**

- York Teaching Hospital NHS FT, York, UK.
- South Warwickshire NHS Foundation Trust, South Warwickshire, UK
- University Hospital Birmingham NHS Foundation Trust, Birmingham, UK.
- Queen Elizabeth Hospital Birmingham, Birmingham, UK.
- Derby Teaching Hospitals NHS FT, Derby, UK.
- Leeds Teaching Hospital, Leeds, UK.
- Royal Wolverhampton Hospitals NHS FT, Wolverhampton, UK.
- University Hospital Southampton NHS FT, Southampton, UK.
- University Hospitals of North Midlands, NHS Trust, Stoke on Trent, UK.
- St. George’s University Hospitals NHS FT, London.
- Bradford Teaching Hospital NHS FT, Bradford, UK.
- Calderdale and Huddersfield NHS FT, Huddersfield, UK.
- Nottingham University Hospitals NHS Trust, Nottingham, UK.

**
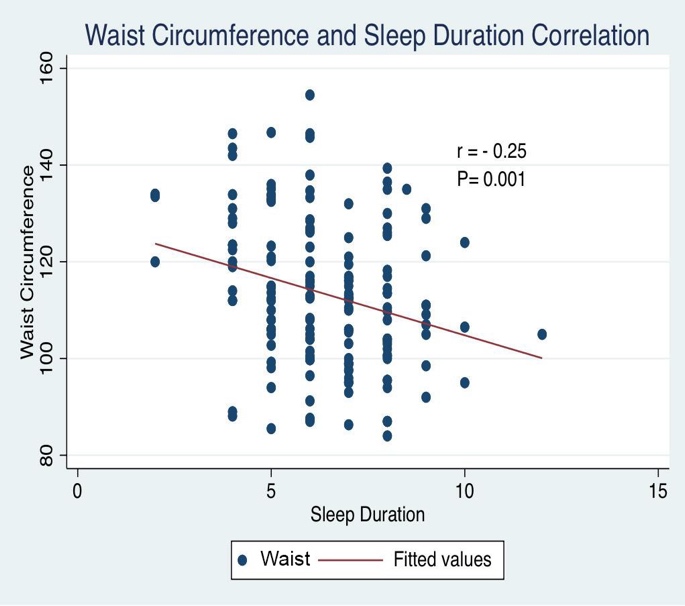
**

**Figure S1: Correlation between sleep duration and adiposity measures, including BMI and waist circumference.**

**Table S1: Baseline characteristics of the study population and by sleep quality.**

Data is presented as n (%), median (IQR), or mean (SD). Analysis was performed using the Chi-square test for categorical variables, the independent t-test for normally distributed variables, and the Mann-Whitney U test for non-normally distributed variables.

|  | **Total**  **(N= 194)** | **Poor Sleep**  **(N= 14§7)** | **Good Sleep**  **(N= 47)** | **P-value** |
| --- | --- | --- | --- | --- |
| **Demographics, n (%)** | | | | |
| **Age (years), mean (SD)** | 61.5 (11.5) | 60.15 (11.1) | 65.91 (11.6) | **0.002** |
| **Gender: male, n (%)** | 117 (60.9) | 85 (57.8) | 32 (71.1) | 0.11 |
| **Ethnicity: White, n (%)** | 162 (83.9) | 122 (82.9) | 40 (86.9) | 0.52 |
| **Smoking and alcohol use, n (%)** | | | | |
| **Smoking (ex/current)** | 111 (57.5) | 85 (58.2) | 26 (55.3) | 0.72 |
| **Alcohol (ex/current)** | 103 (53.7) | 74 (50.7) | 29 (63.0) | 0.14 |
| **Diabetes duration and medication used, median (IQR), n (%)** | | | | |
| **Diabetes duration (years), median (IQR)** | 13 (6 - 19) | 12 (6 - 19) | 16.5 (6 - 19) | 0.15 |
| **Insulin, n (%)** | 99 (51.6) | 80 (54.8) | 19 (41.3) | 0.11 |
| **GLP-1 (glucagon like peptide 1) agonist, n (%)** | 33 (18.6) | 26 (19.4) | 7 (16.3) | 0.64 |
| **Lipid lowering agents (statin), n (%)** | 135 (72.6) | 1045 (73.8) | 31 (69.2) | 0.52 |
| **Anti-hypertensive (ACE inhibitor), n (%)** | 84 (45.9) | 62 (44.9) | 22 (48.9) | 0.64 |
| **Blood Pressure (mmHg), mean (SD)** | | | | |
| **Systolic** | 132.91 (16.0) | 132.51 (16.7) | 134.03 (13.9) | 0.38 |
| **Diastolic** | 75.61 (10.9) | 74.96 (10.8) | 77.41 (10.9) | 0.17 |
| **Biochemistry Measures** |  |  |  |  |
| **Total cholesterol (mmol/L)** | 4.12 (1.1) | 4.15 (1.1) | 4.02 (1.0) | 0.48 |
| **HbA1c (mmol/mol)** | 65.68 (21.2) | 68.00 (21.7) | 58.15 (17.5) | **0.008** |
| **Adiposity Measures, mean (SD)** |  |  |  |  |
| **BMI (kg/m^2^)** | 33.69 (7.7) | 34.64 (7.7) | 30.86 (6.9) | **0.002** |
| **Obesity (BMI ≥30 kg/m^2^)** | 116 (63.4) | 96 (70.1) | 20 (43.5) | **0.001** |
| **Waist Circumference** | 112.93 (15.1) | 114.98 (14.1) | 107.04 (16.2) | **0.002** |
| **High Waist** | 129 (66.5) | 104 (70.8) | 25 (53.2) | **0.02** |

eGFR = glomerular filtrate rate; GLP-1 = glucagon-like peptide 1; HbA1c = glycated haemoglobin.

**Table S2: Assessing the association between sleep quality and adiposity measures, including BMI and waist circumference, based on baseline cross-sectional analysis using multiple linear regression.**

| **Model** | **R2** | **Coefficient** | **95% CI** | **P Value** |
| --- | --- | --- | --- | --- |
| **BMI** | | | | |
| **Unadjusted** | 0.07 | 0.57 | 0.28 - 0.86 | <0.0001 |
| **Model^1^** | 0.27 | 0.42 | 0.11 - 0.73 | 0.008 |
| **Model^2^** | 0.30 | 0.41 | 0.11 - 0.71 | 0.008 |
| **Waist Circumference** | | | | |
| **Unadjusted** | 0.09 | 1.20 | 0.59 - 1.82 | <0.0001 |
| **Model^1^** | 0.29 | 1.37 | 0.70 - 2.03 | <0.0001 |
| **Model^2^** | 0.30 | 1.31 | 0.64 - 1.98 | <0.0001 |

BMI = body mass index; CI = confidence interval.

Model^1^ is adjusted for sleep quality, age, ethnicity, gender, duration of diabetes, insulin use, GLP-1 receptor use, smoking status, and alcohol use.

Model^2^ is adjusted for sleep quality, age, ethnicity, gender, diabetes duration, insulin use, GLP-1 receptor use, smoking status, alcohol use, and OSA.

**Table S3: Relationship between sleep categories (sleep vs normal) and adiposity measures.**

|  | baseline | Follow-up | p-value | | Baseline | Follow-up | p-value |
| --- | --- | --- | --- | --- | --- | --- | --- |
|  | **Short Sleep**  **(N=37)** | | |  | **Normal Sleep**  **(N=44)** | |  |
| BMI, mean (SD) | 34.1 (6.7) | 34.68 (7.19) | | 0.33 | 30.8 (5.7) | 30.2 (5.6) | 0.10 |
| Missing, n | 4 | 7 | |  | 2 | 13 |  |
| Waist Circumference, mean (SD) | 117.7 (16.3) | 119.9 (16.3) | | 0.16 | 108.1 (14.2) | 108.3 (14.6) | 0.83 |
| Missing, n | 0 | 6 | |  | 1 | 12 |  |
| Neck Circumference, mean (SD) | 43.5 (4.9) | 43.7 (4.3) | | 0.4 | 41.3 (4.4) | 41.5 (4.6) | 0.65 |
| Missing, n | 0 | 6 | |  | 1 | 12 |  |
| Short sleep (N=37) | **Short Sleep**  **Who remained as short sleep**  **(N=24)** | | |  | **Short Sleep**  **Who changed to normal sleep**  **(N=13)** | |  |
| BMI, mean (SD) | 35.4 (6.9) | 35.9 (7.4) | | 0.36 | 31.3 (5.4) | 31.8 (6.1) | 0.70 |
| Missing, n | 3 | 3 | |  | 4 | 1 |  |
| Waist Circumference, mean (SD) | 119.1 (17.3) | 120.9 (16.9) | | 0.35 | 114.3 (14.1) | 117.7 (15.6) | 0.28 |
| Missing, n | 0 | 2 | |  | 0 | 3 |  |
| Neck Circumference, mean (SD) | 43.7 (5.1) | 44.1 (4.6) | | 0.52 | 42.8 (4.9) | 42.9 (3.5) | 0.80 |
| Missing, n | 0 | 2 | |  | 0 | 4 |  |
| Normal sleep (N=44) | **Normal sleep**  **Who remind as normal sleep**  **(N=33)** | | |  | **Normal Sleep**  **Who changed to short sleep**  **(N=11)** | |  |
| BMI, mean (SD) | 30.9 (6.2) | 30.3 (6.4) | | 0.06 | 30.1 (3.9) | 29.8 (1.7) | 0.77 |
| Missing, n | 2 | 9 | |  | 0 | 4 |  |
| Waist Circumference, mean (SD) | 109.5 (14.8) | 109.7 (15.6) | | 0.83 | 103.5 (11. 9) | 103.5 (9.9) | 0.97 |
| Missing, n | 1 | 8 | |  | 0 | 4 |  |
| Neck Circumference, mean (SD) | 41.5 (4.5) | 41.7 (4.9) | | 0.55 | 40.5 (4.2) | 40.4 (3.4) | 0.92 |
| Missing, n | 1 | 8 | |  | 0 | 4 |  |

Note: The analysis included data from patients who had both baseline and follow-up for the groups’ short sleep and normal sleep
